# Publisher Correction: EMT cells increase breast cancer metastasis via paracrine GLI activation in neighbouring tumour cells

**DOI:** 10.1038/s41467-018-07168-z

**Published:** 2018-11-12

**Authors:** Deepika Neelakantan, Hengbo Zhou, Michael U. J. Oliphant, Xiaomei Zhang, Lukas M. Simon, David M. Henke, Chad A. Shaw, Meng-Fen Wu, Susan G. Hilsenbeck, Lisa D. White, Michael T. Lewis, Heide L. Ford

**Affiliations:** 1 Department of Pharmacology, University of Colorado–Denver, 12800 East 19th Avenue, Room P18-6115, Aurora, Colorado 80045 USA; 2 Molecular Biology Program, University of Colorado Anschutz Medical Campus, Aurora, Colorado 80045 USA; 3 Cancer Biology Program, University of Colorado Anschutz Medical Campus, Aurora, Colorado 80045 USA; 4 Integrated Physiology Program, University of Colorado Anschutz Medical Campus, Aurora, Colorado 80045 USA; 5 Lester and Sue Smith Breast Center, Baylor College of Medicine, Houston, Texas 77030 USA; 6 Institute of Computational Biology, Helmholtz Zentrum München (GmbH), 85764 Neuherberg, Germany; 7 Department of Molecular and Human Genetics, Baylor College of Medicine, Houston, Texas 77030 USA; 8 Department of Medicine, Baylor College of Medicine, Houston, Texas 77030 USA; 9 Departments of Molecular and Cellular Biology and Radiology, Baylor College of Medicine, Houston, Texas 77030 USA

Correction to: *Nature Communications* 10.1038/ncomms15773, published online 12 June 2017

This Article contains an error in Fig. 2. In panel a, the second lane of the western blot should have been labelled as ‘siNT’. A correct version of Fig. 2a appears below; the error has not been fixed in the original Article.

**Fig. 2:**
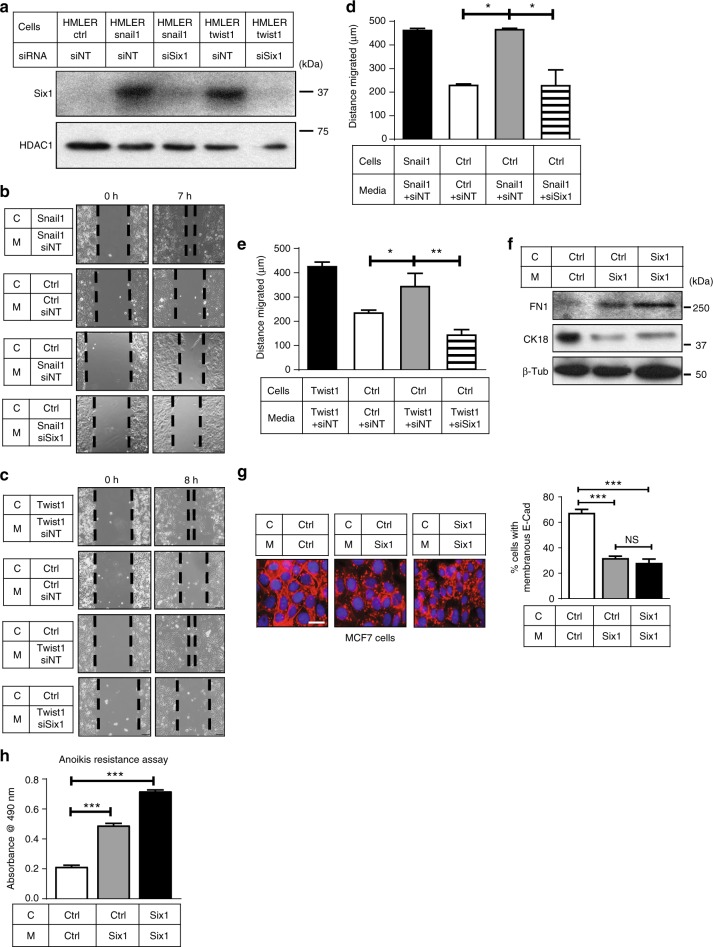
▓

