# Supplementary material for: EMT cells increase breast cancer metastasis via paracrine GLI activation in neighbouring tumour cells
Source: Nat Commun. 2017 Jun 12;8:15773. doi: 10.1038/ncomms15773 (PMC5472791; doi:10.1038/ncomms15773)
Supplement: Supplementary Information — Supplementary Figures and Supplementary Tables [file ncomms15773-s1.pdf]

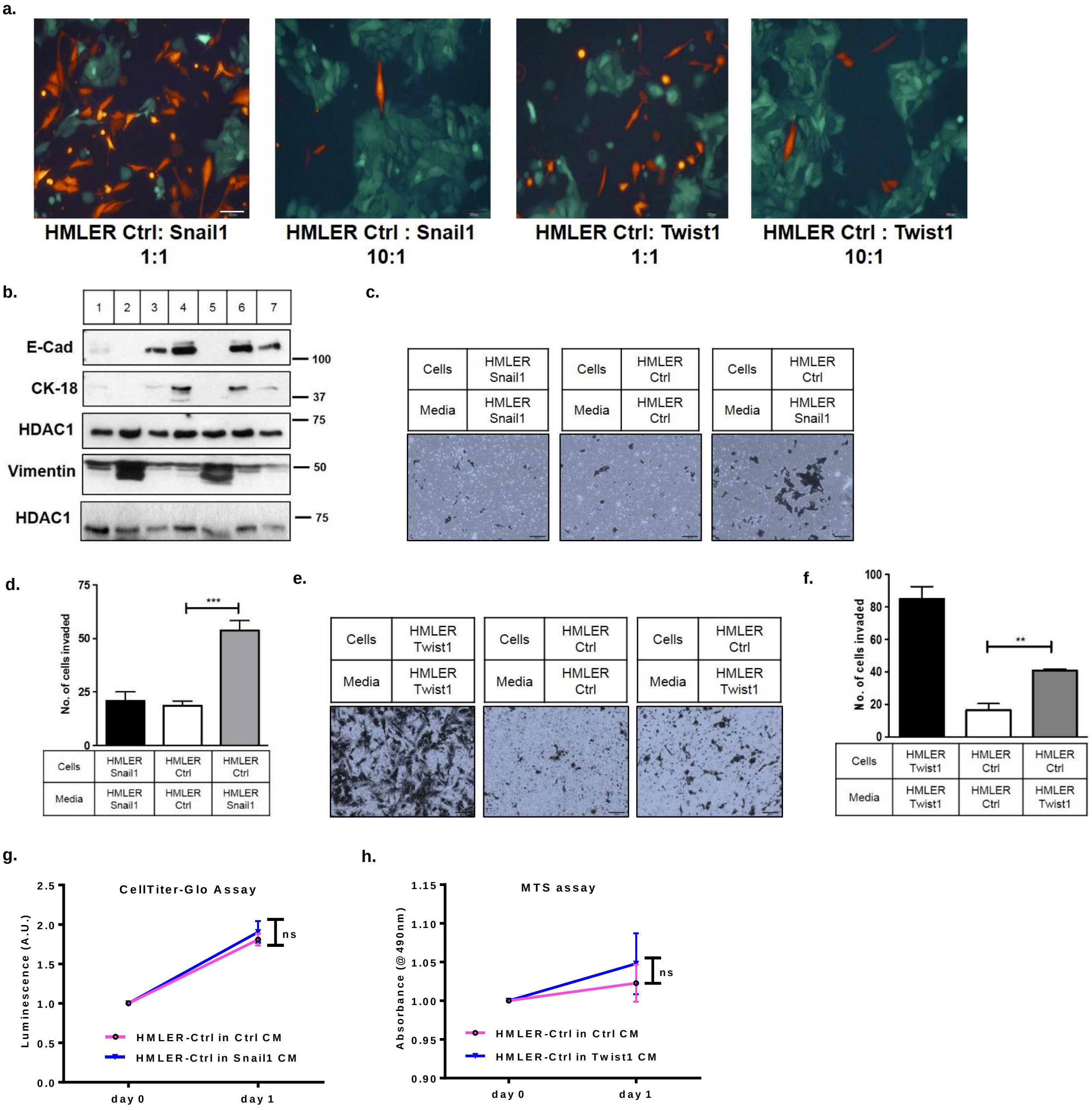

Supplementary Figure 1. Snail1 and Twist1 non-cell autonomously increase invasion of HMLER-Ctrl cells without altering EMT markers

(**a**) Representative image of HMLER-Ctrl cells co-cultured with HMLER-Snail1/Twist1 cells at 1:1 and 10:1 ratio (day 11 of 14).

(**b**) Representative Western blot analyses of whole cell lysates (WCLs) from GFP^+ or $tRFP^+$ sorted from co-culture experiments at day 14. (HDAC1 – loading control), n≥3.

Lanes: (1) GFP^+HMLER-Ctrl cells, (2) $tRFP^+$ HMLER-Snail1 cells, (3, 4) GFP^+HMLER-Ctrl cells from (3) 1:1 and (4) 10:1 co-culture with HMLER-Snail1 cells, (5) $tRFP^+$ HMLER-Twist1 cells, (6, 7) GFP^+HMLER-Ctrl cells from (6) 1:1 and (7) 10:1 co-culture with HMLER-Twist1 cells.

(**c,e**) Representative 18hr invasion assays of HMLER-Snail1/Twist1 and HMLER-Ctrl cells in indicated CM.

(**d,f**) Quantitation of invasion assays in panel **c** and **e**.

(**g,h**) Representative proliferation assay by MTS (**g**) and CellTiter-Glo (**h**) for HMLER-Ctrl cells in indicated CM.

Scale bar-100µm; S.E.M shown, One way ANOVA with Tukey post test in **d,f**; n≥6; RM Two-way ANOVA in **g,h**; ns- not significant, **p value<0.001, ***p value<0.0001

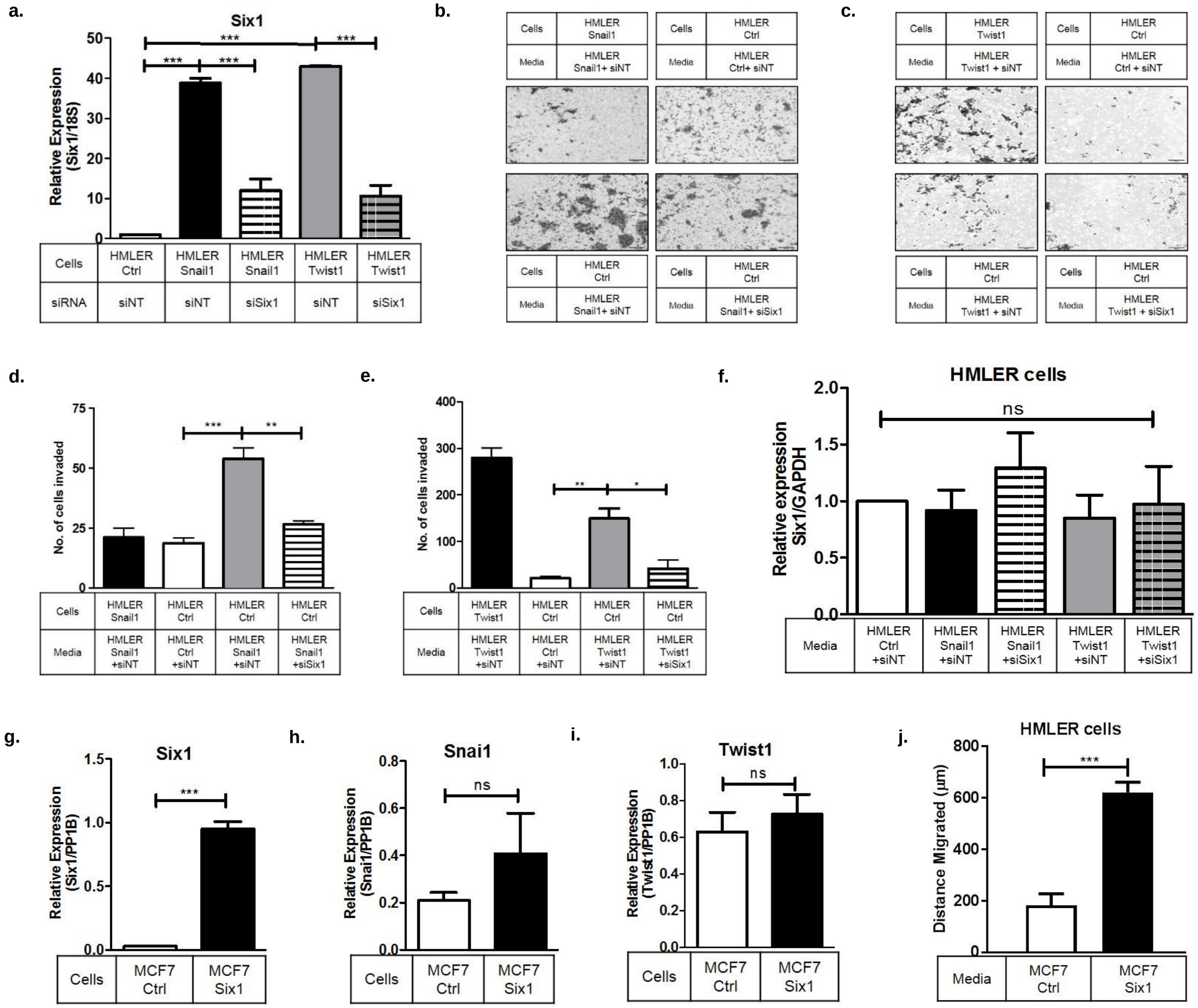

Supplementary Figure 2. Six1 is upregulated downstream of Snail1 and Twist1 in HMLER cells and only Six1 is overexpressed in MCF7 cells

(**a**) qRT-PCR analyses of HMLER-Ctrl, Snail1 and Twist1 cells show that Six1 is upregulated downstream of Snail1 and Twist1 and is knocked down in cells transfected with 150nM of siSix1 compared to a non-targeting siRNA pool (siNT).
(**b,c**) Representative 18hr invasion assay of HMLER-Ctrl cells in CM from HMLER-Snail1 or Twist1 cells +/- siSix1.
(**d,e**) Quantitation of cell invasion in panel **b** and **c** respectively.
(**f**) qRT-PCR analyses of Six1 levels in HMLER-Ctrl cells cultured in CM from HMLER-Ctrl or HMLER-Snail1/Twist1 +/- siSix1.
(**g-i**) qRT-PCR analyses of 3 clones each of MCF7-Ctrl and MCF7-Six1 cells (combined data shown for each group). Gene expression normalized to 18s, GAPDH or PP1B.
(**j**) 7hr migration assay of HMLER-Ctrl cells in CM from MCF7-Ctrl and Six1 cells (combined data shown for >3 experiments).
S.E.M shown, One way ANOVA with Tukey post test on compiled experiments n≥3 in **a,d-f;** two tailed unpaired t-test in **g-j**, n=3; ns- not significant, *p<0.05, **p<0.001, ***p<0.0001

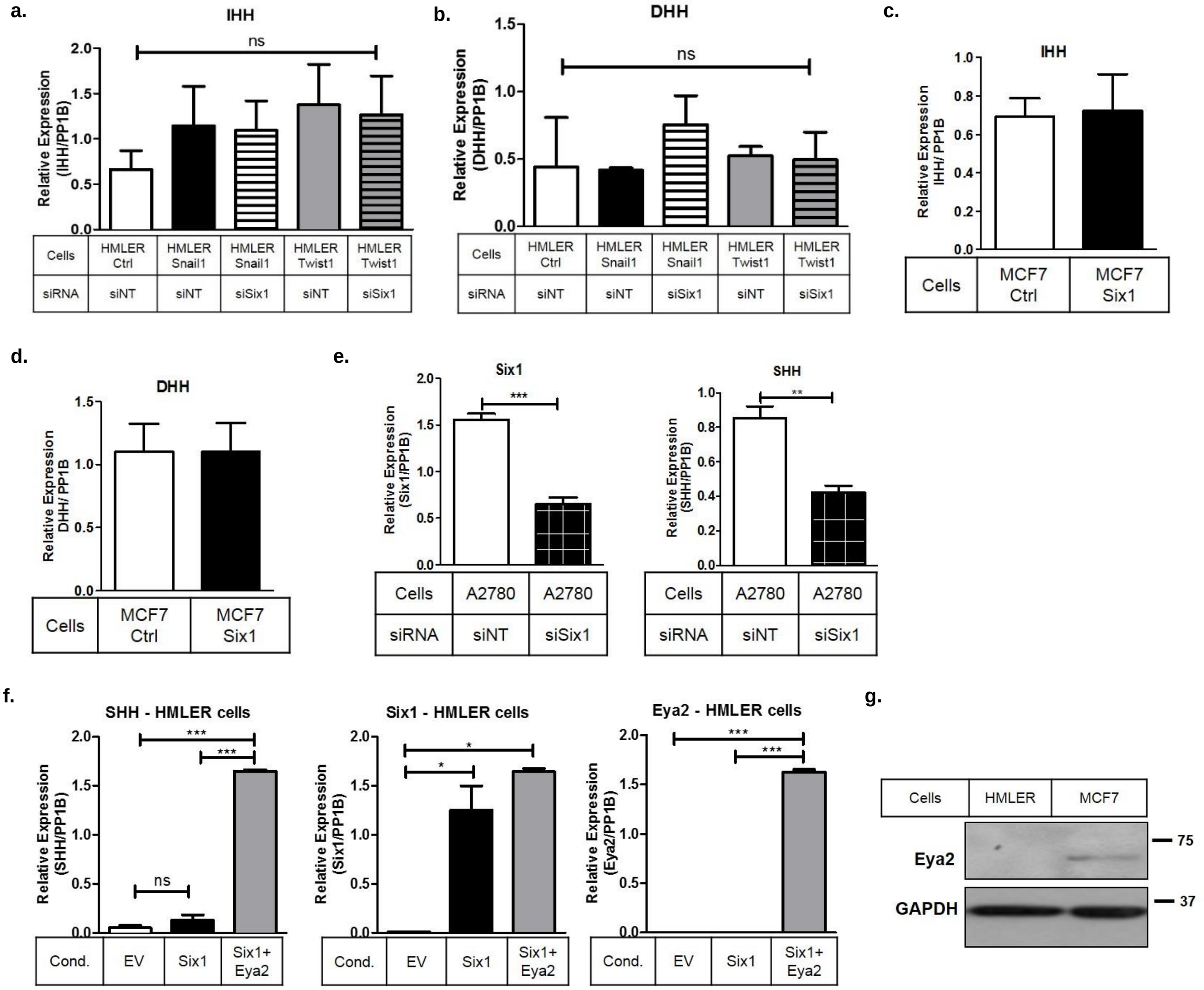

Supplementary Figure 3. Hh ligand levels are differentially regulated downstream of Snail1, Twist1 and Six1

(**a,b**) qRT-PCR analyses in HMLER-Ctrl, Snail1 and Twist1 cells transfected with 150nM of siNT or siSix1.
(**c,d**) qRT-PCR analyses in combination of 3 clones each of MCF7-Ctrl and MCF7-Six1 cells.
(**e**) qRT-PCR analyses in A2780 cells transfected with 100nM of siNT or siSix1.
(**f**) qRT-PCR analyses in HMLER-Ctrl cells transfected with 1µg of Six1 and/or Eya2. EV-Empty vector.
(**g**) Western blot analyses of whole cell lysates from HMLER and MCF7 cells showing endogenous levels of Eya2.
Gene expression normalized to PP1B. S.E.M shown, compiled experiments $n \geq 3$. One way ANOVA with Tukey post test in (**a,b,f**), two tailed unpaired t-test in (**c-e**). ns – not significant; *p value<0.05, **p<0.001, ***p<0.0001

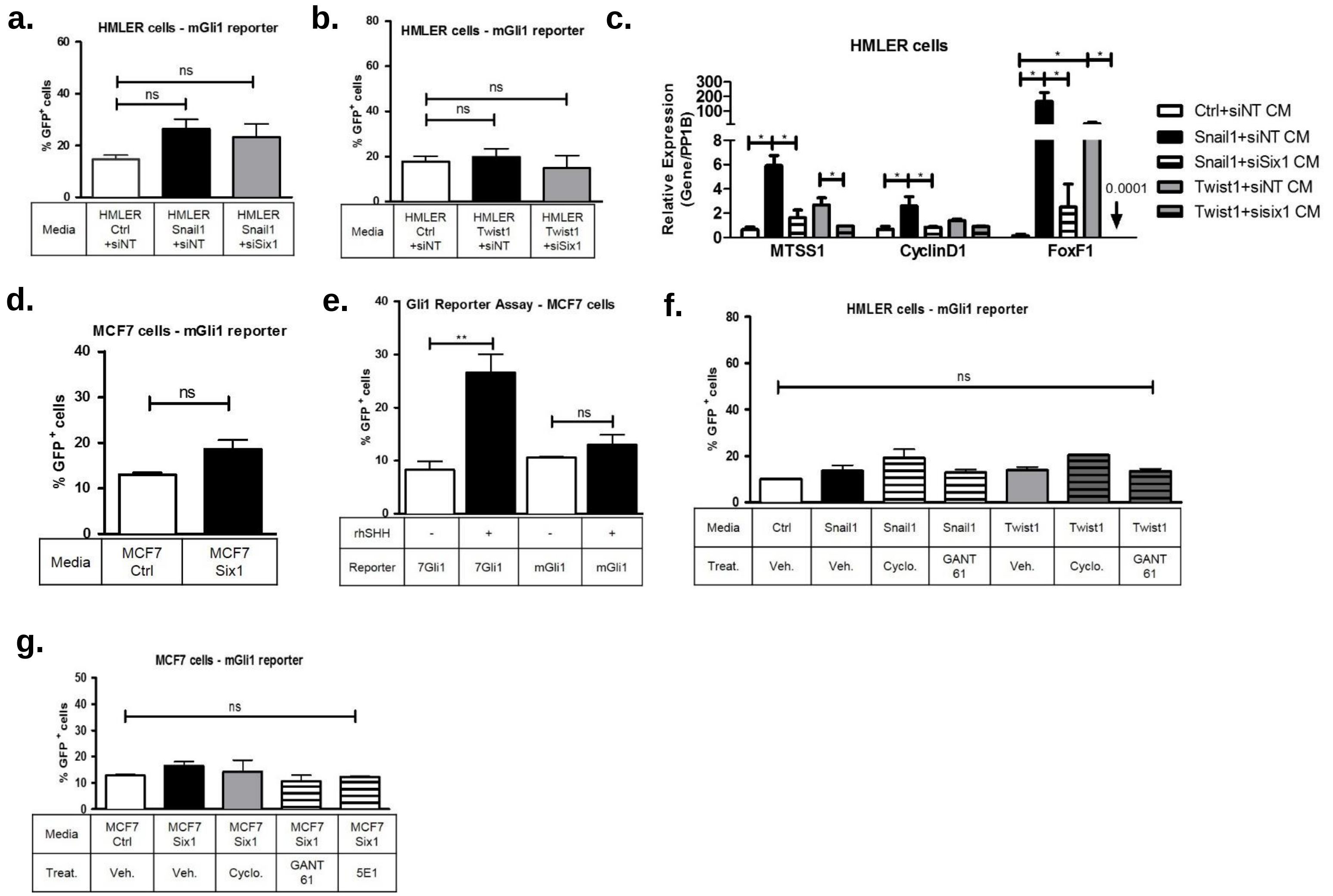

Supplementary Figure 4. Hh/Gli signaling is specifically activated in the presence of CM from cells expressing EMT-TFs

(a,b) mutant (m)-Gli1-GFP reporter assay in HMLER cells cultured in CM from HMLER-Ctrl and (**a**) HMLER-Snail1+/-siSix1 cells or (**b**) HMLER-Twist1+/-siSix1 cells.

(**c**) qRT-PCR analyses of Hh pathway target genes in HMLER-Ctrl cells in different CM; gene expression normalized to PP1B.

(**d)** m-Gli1-GFP reporter assay in MCF7-Ctrl cells cultured in MCF7-Ctrl/Six1 CM.

(**e**) 7-Gli1 and m-Gli1-GFP reporter assays in MCF7-Ctrl cells with addition of 1µg/ml of rhSHH to media.

(f,g) m-Gli1-GFP reporter assay in (**f**) HMLER cells in CM from HMLER-Ctrl, Snail1 or Twist1 cells, and in (**g**) MCF7-Ctrl cells in MCF7-Ctrl/Six1 CM treated with indicated drug/antibodies.

All Gli1-GFP reporter assays are represented as %GFP$^+$ cells.

S.E.M shown, compiled experiments n≥2 (**c**),n=3 (with different sets of CM). One way ANOVA with Tukey post test in all cases. ns – not significant; *p value<0.05, ***p value<0.0001

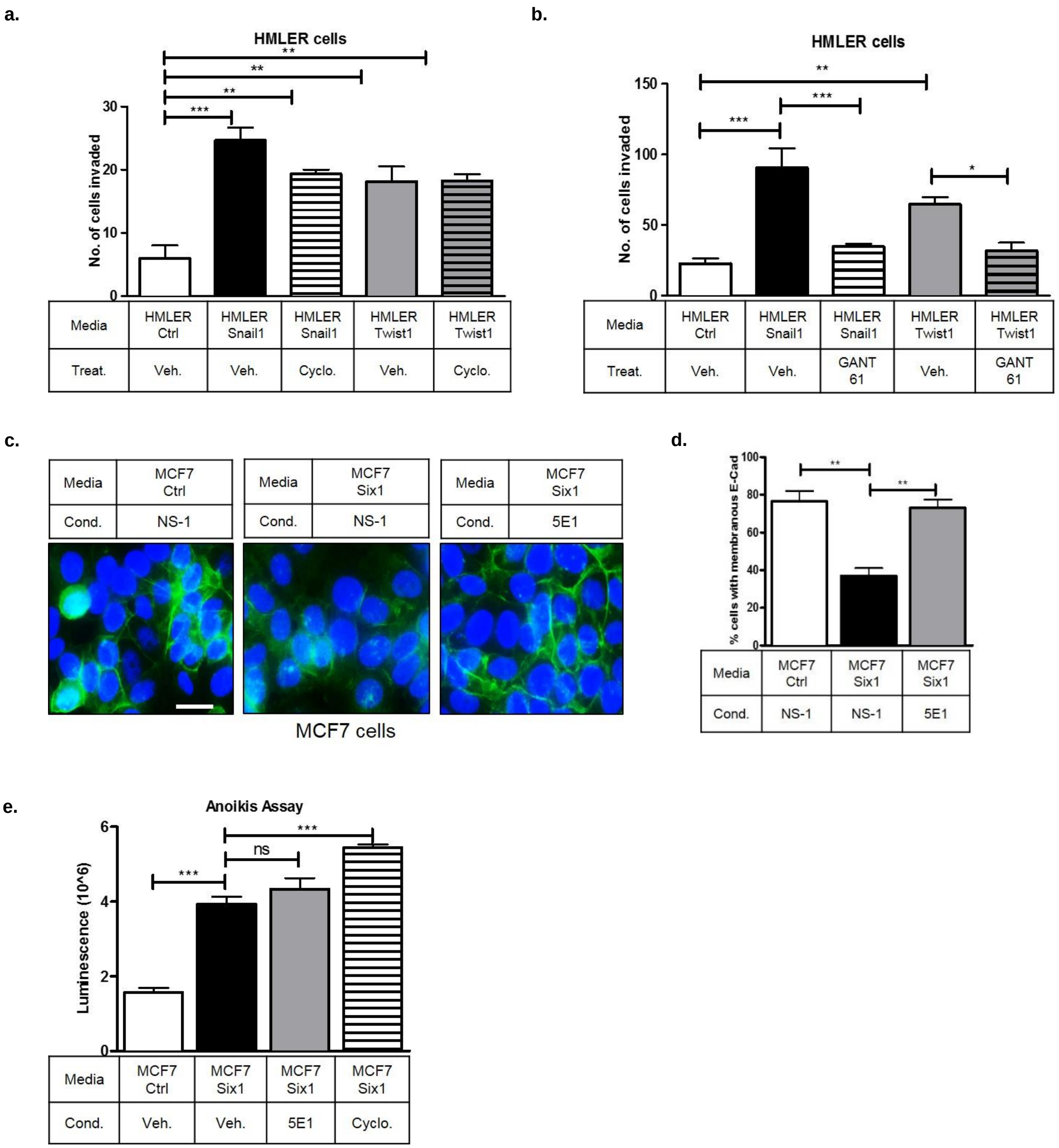

Supplementary Figure 5. Hh pathway inhibition via upstream vs downstream inhibitors differentially regulates non-cell autonomous phenotypes dependent on context.
(a,b) 16-18hr invasion assays of HMLER-Ctrl cells cultured in HMLER-Snail1/Twist1 CM treated with **(a)** cyclopamine or **(b)** GANT61 or corresponding vehicle control.
(c) Representative ICC of E-cadherin (green) in MCF7-Ctrl cells cultured in CM from MCF7-Ctrl and Six1 cells, treated with 5E1 and control NS-1 antibody for 48hrs (Dapi, blue), scale bar – 20µm.
(d) Quantitation of % membranous E-cadherin in panel **c**, cell counted per condition ≥ 45-60.
(e) Anoikis resistance assay in MCF7-Ctrl cells cultured in indicated CM +/- cyclopamine or 5E1 for 24hrs.
S.E.M shown, n≥3. One way ANOVA with Tukey post test in all cases. ns – not significant; *p<0.05, **p<0.001, ***p<0.0001

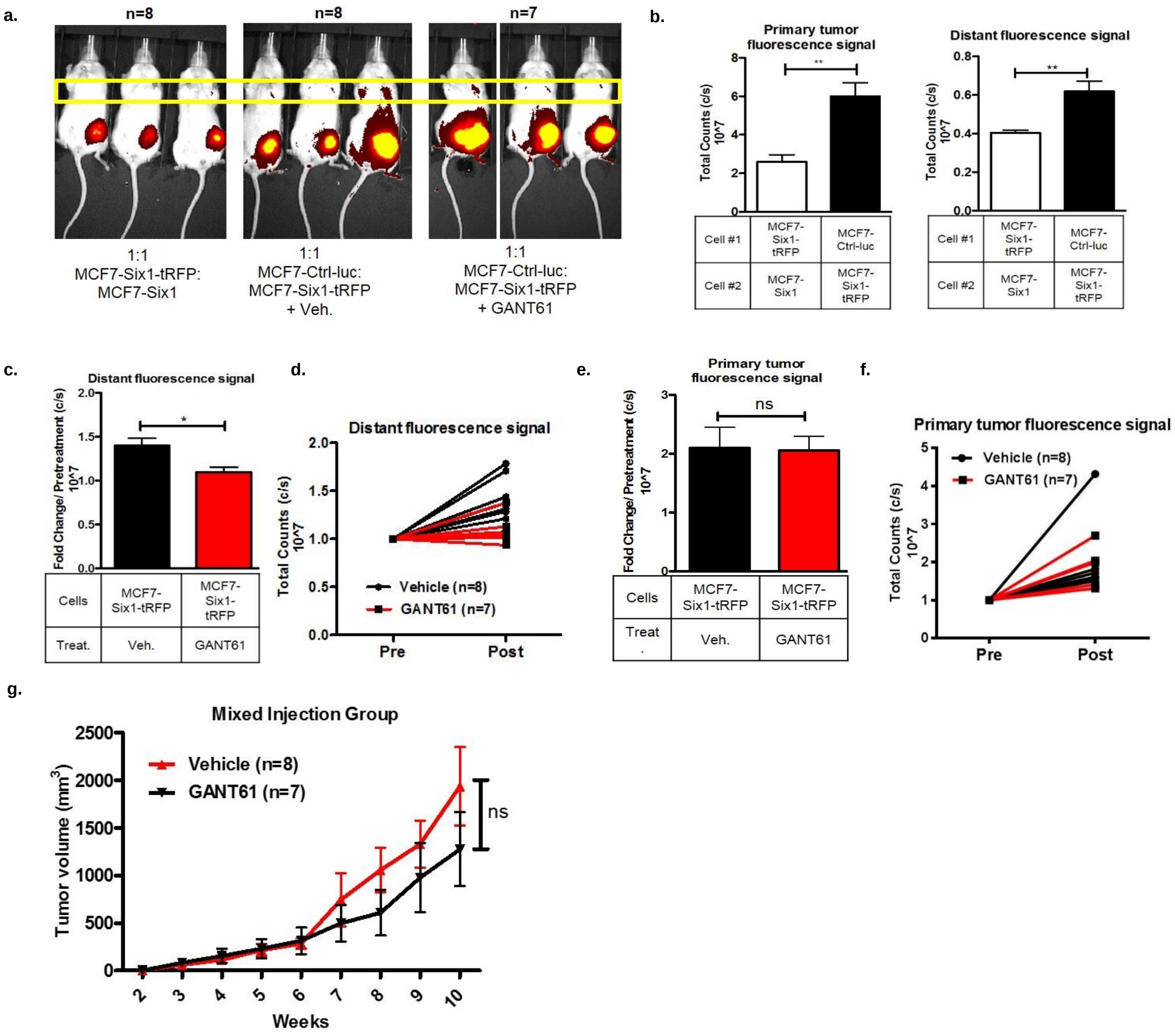

Supplementary Figure 6. Six1-expressing EMT cells benefit from inter-clonal cooperation with non-EMT non-Six1 expressing cells.

(**a**) Representative fluorescent images of NOG/SCID mice at same time point (corresponding to similar tumor volumes) with MCF7-Six1-tRFP "singly" injected tumors and GANT61 or vehicle treated mixed injection groups.

(**b**) Quantitation of red fluorescent signal (EMT cells) from primary tumor and distant sites (in lymph nodes/lungs - yellow boxed region in **a** representing MCF7-Six1 cells) in MCF7-Six1-tRFP and mixed tumor groups represented as c/s, counts per second.

(**c,e**) Quantitation of fluorescent signal (representing MCF7-Six1 EMT cells) from (**c**) distant sites and (**e**) primary tumor in mixed tumors groups treated with vehicle or GANT61, represented as fold change over pre-treatment signal.

(**d,f**) Normalized fluorescent signal (MCF7-Six1 cells) from (**d**) distant sites and (**f**) primary tumors of individual mice pre- and post-treatment.

(**g**) Overall tumor volumes of mice that received vehicle or GANT61 treatment over the course of the experiment, measured by calipers.

S.E.M shown, two tailed unpaired t-test for **a-f**; Two-way ANOVA followed by Bonferroni post test for **g;** ns- not significant; *p value<0.05, **p value<0.001

Fig 2a.

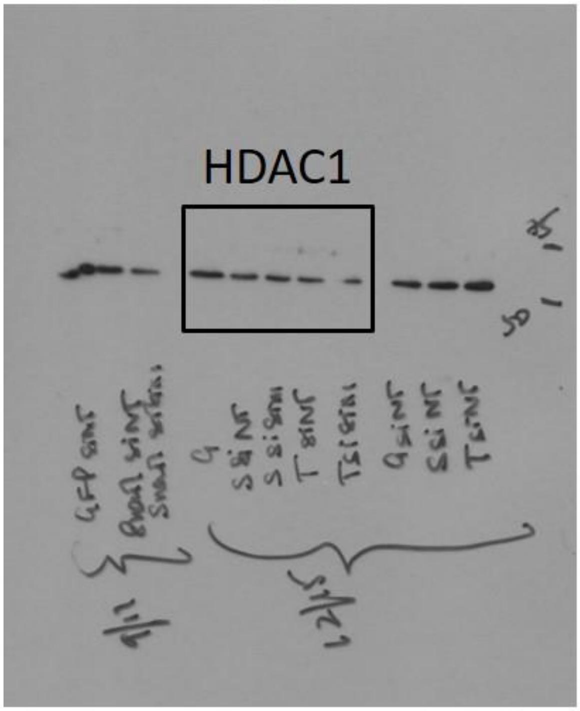

G – HMLER-Ctrl
S – HMLER-Snail1
T – HMLER-Twist1

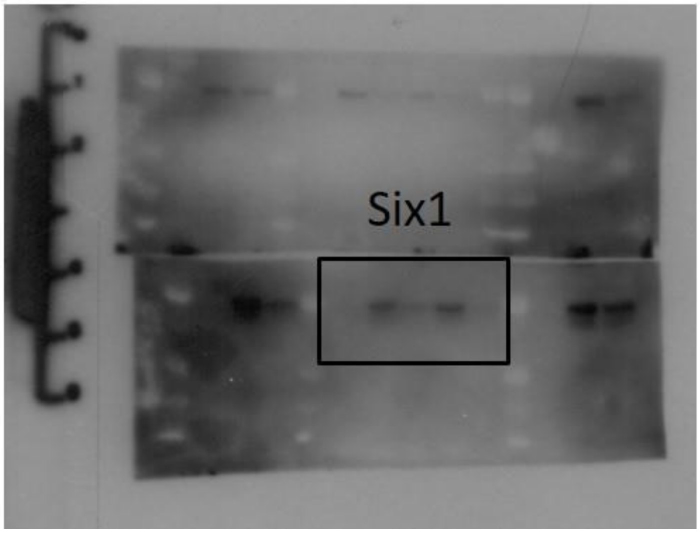

Supplementary Figure 7. Uncropped blots for western blots analyses in the main figures

Fig 2f.

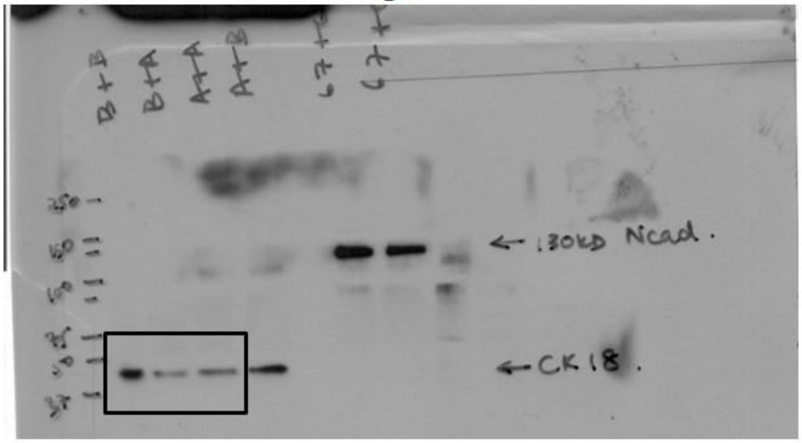

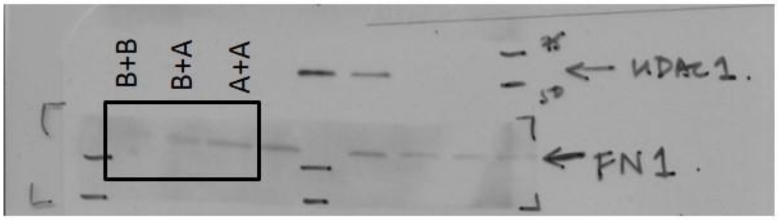

B – MCF7-Ctrl
A – MCF7-Six1
B+B – Ctrl cells in Ctrl CM
B+A – Ctrl cells in Six1 CM
A+A – Six1 cells in Six1 CM

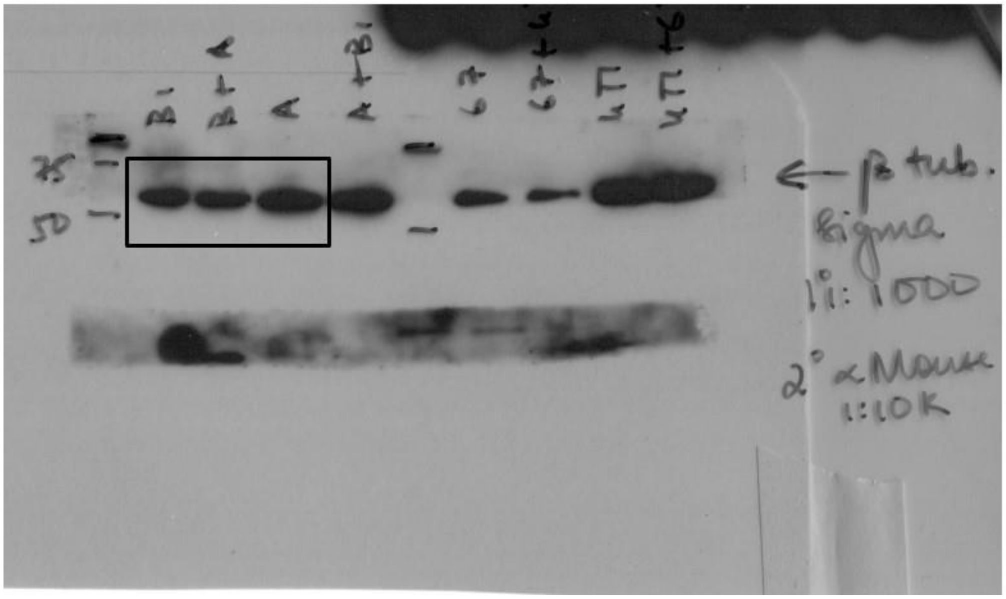

Supplementary Fig. 7 continued

Fig 3d.

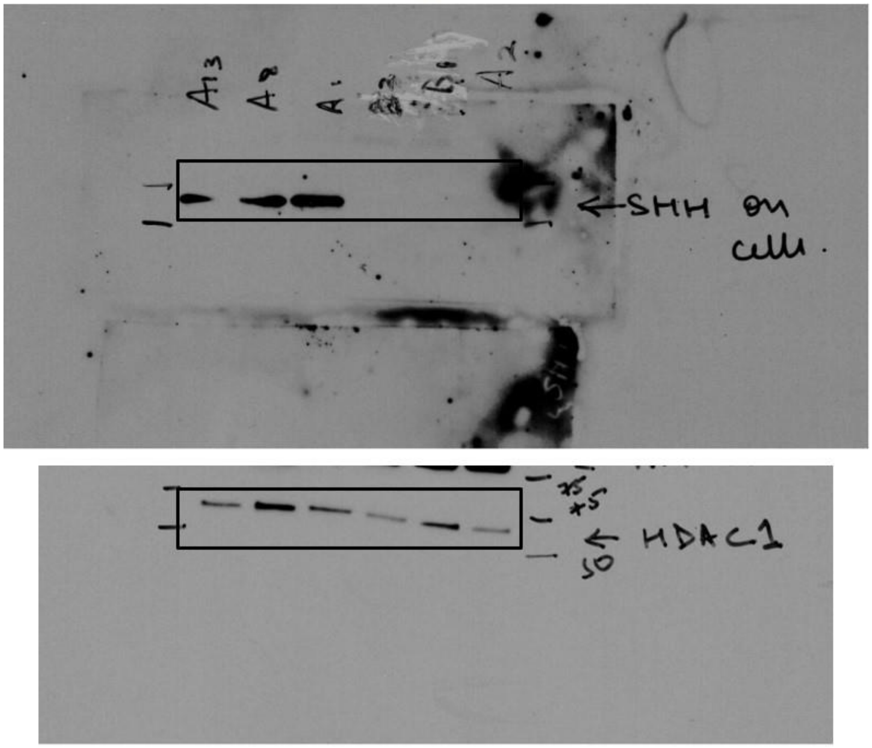

B – MCF7-Ctrl clones
A – MCF7-Six1 clones

Blot is reversed in paper

Fig 5e.

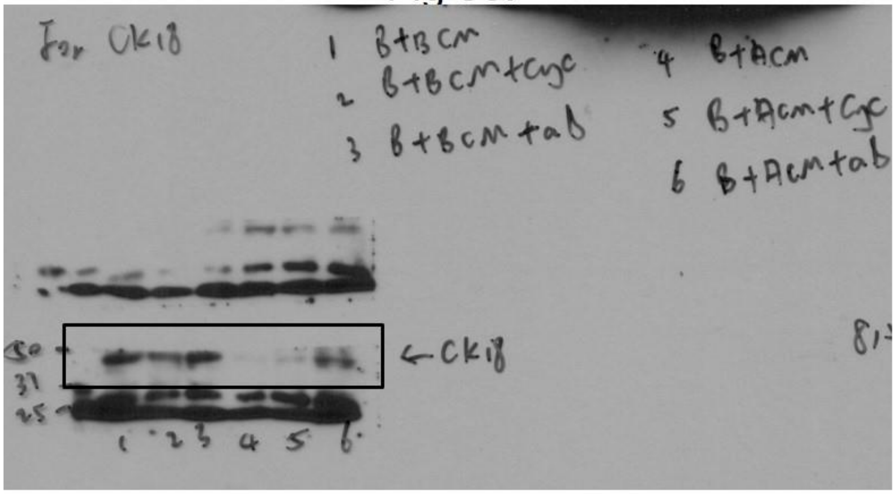

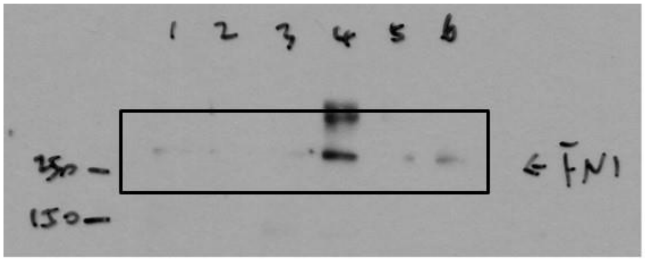

B – MCF7-Ctrl
A – MCF7-Six1
B+B – Ctrl cells in Ctrl CM
B+A – Ctrl cells in Six1 CM
A+A – Six1 cells in Six1 CM
Cyc – cyclopamine
Ab – 5E1

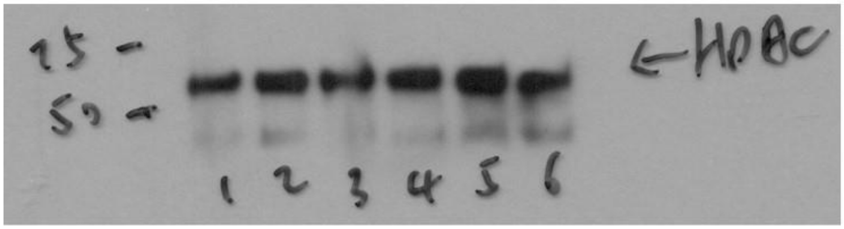

Supplementary Fig. 7 continued

Fig 5g.

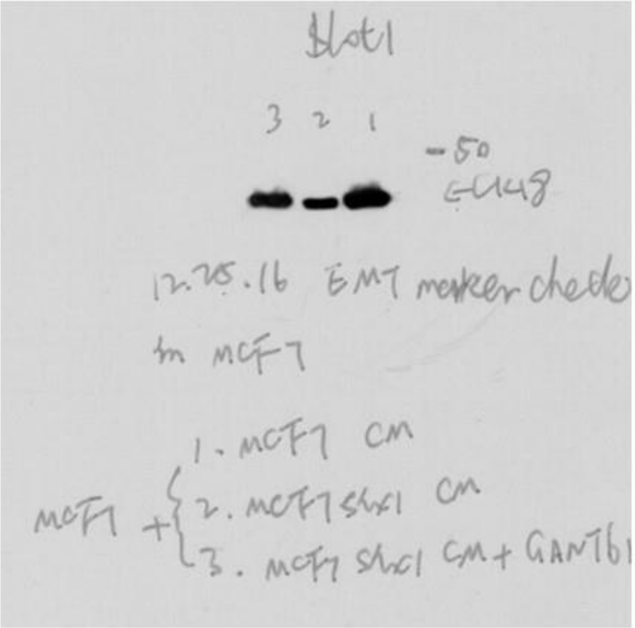

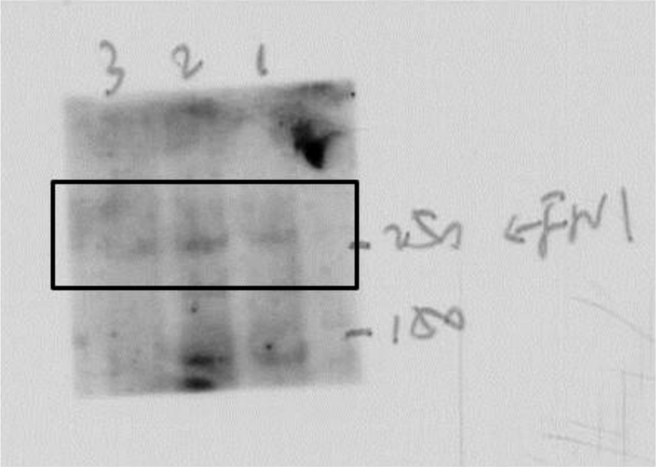

B – MCF7-Ctrl
A – MCF7-Six1
Blot is reversed in paper

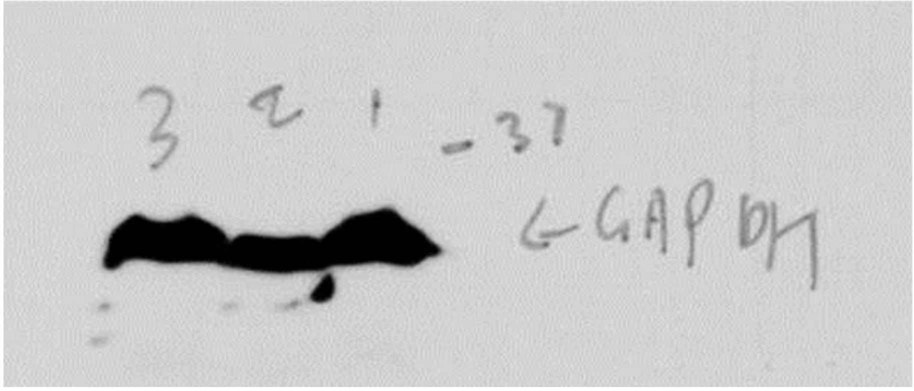

Supplementary Fig 1b.

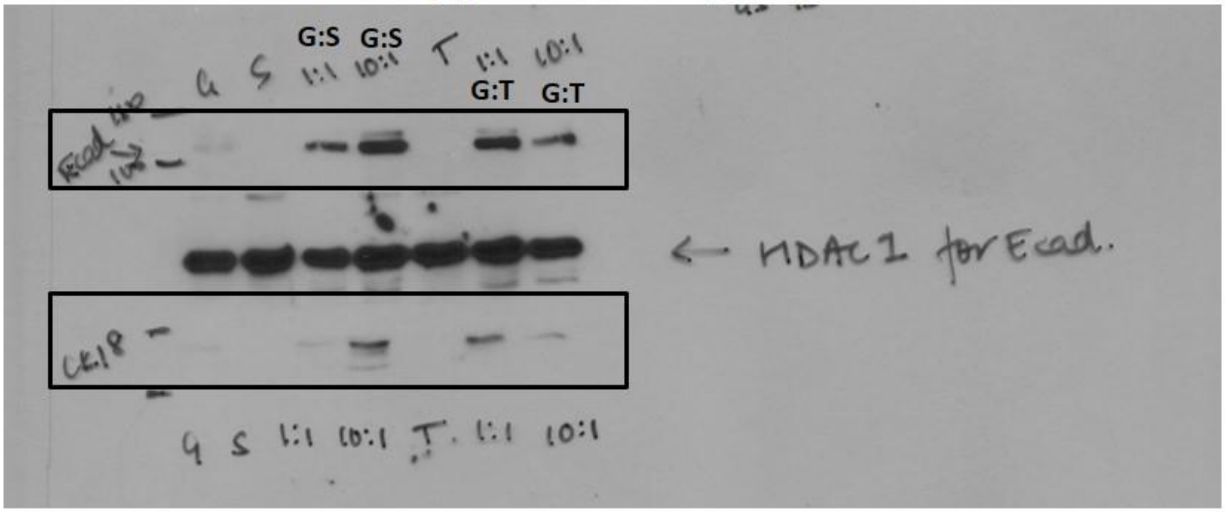

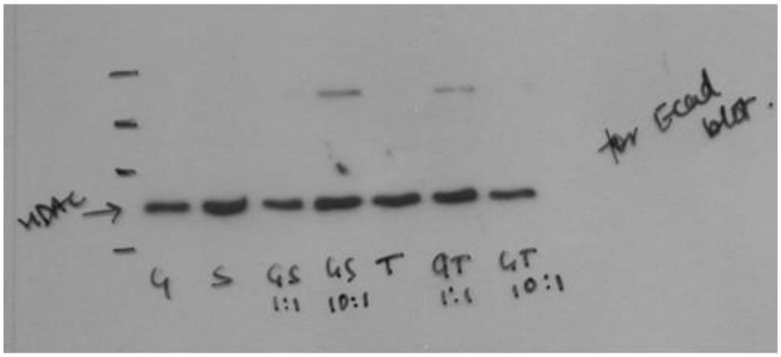

G- HMLER-Ctrl
S – HMLER-Snail1
T- HMLER-Twist1
1:1, 1:10 – ratio of G to S/T cells

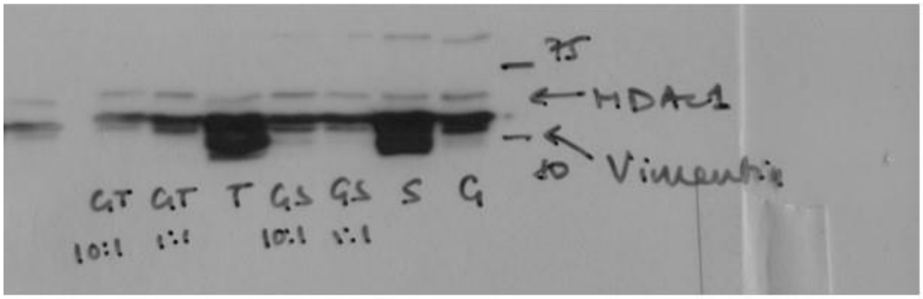

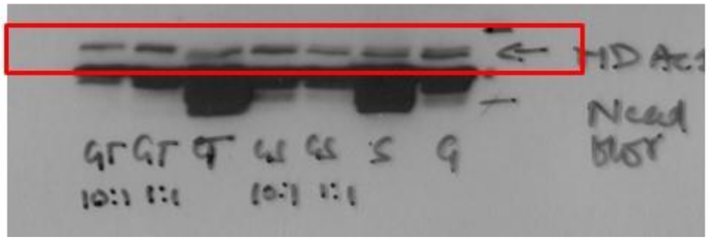

Blot is reversed in paper

Supplementary Fig 3g.

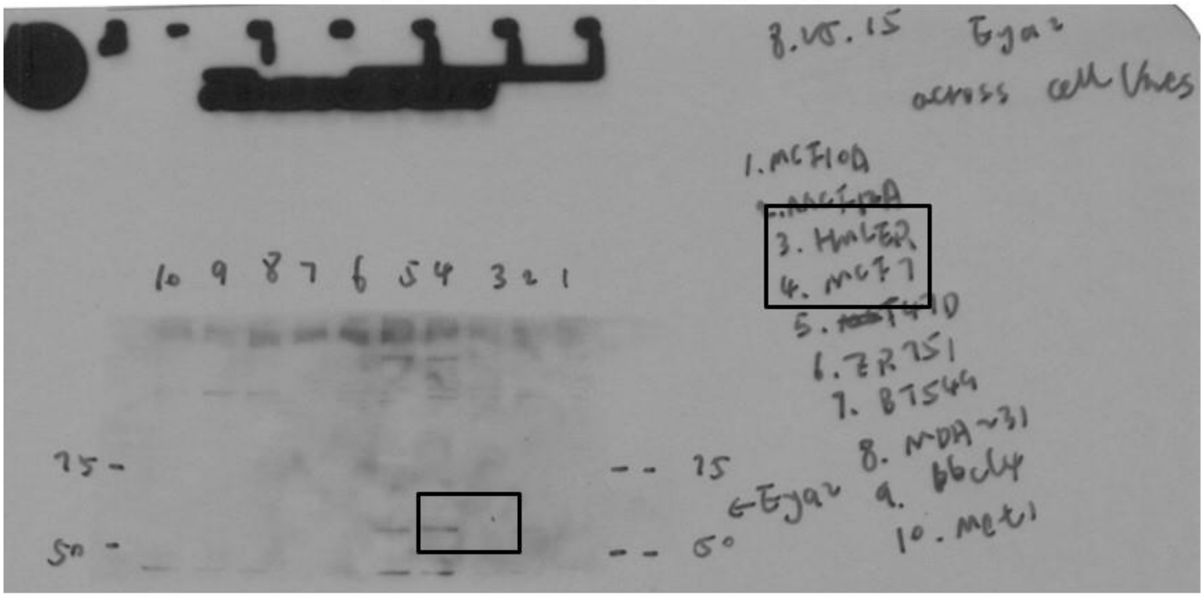

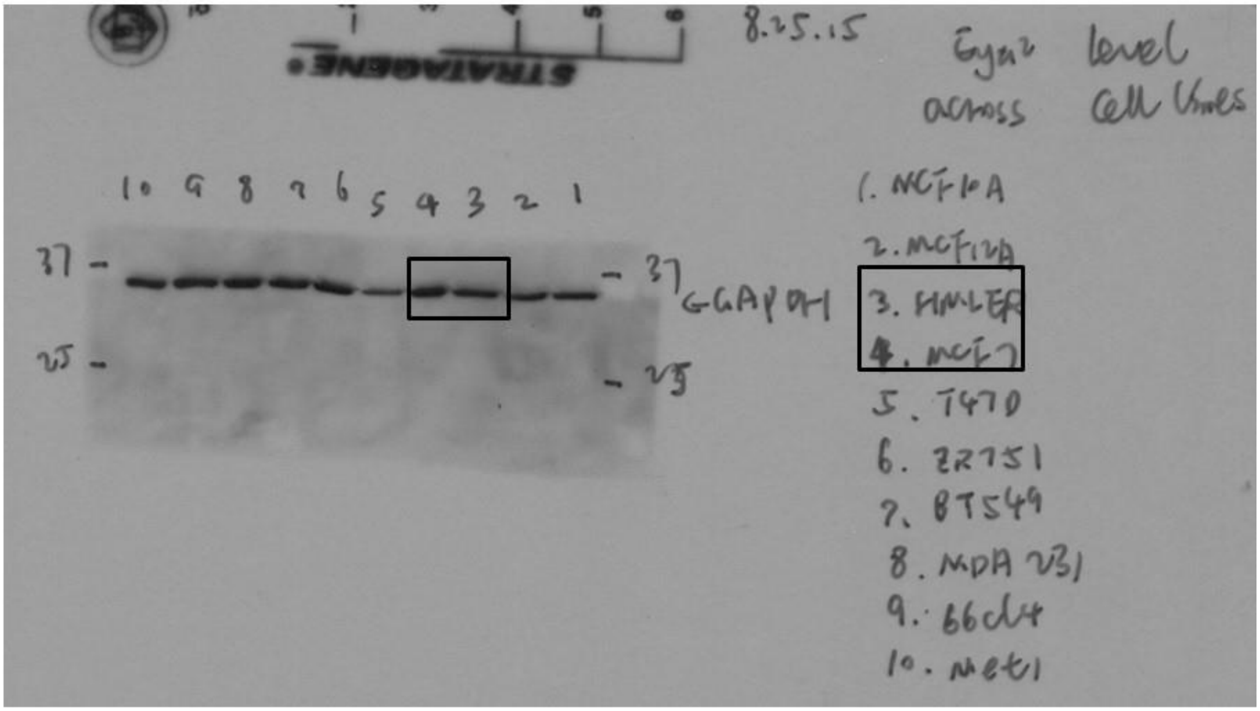

Blot is reversed in paper

Supplementary Table 1.

Combination	Prognosis	Subtype/Grade	p value
Six1, Gli1	DMFS	Luminal A	0.0121
	DMFS	ER+ LN negative tumors	0.01923
	DMFS	LN negative tumors	0.00919
	DMFS mixed	PAM50-Luminal A	0.03606
	DMFS mixed	LN negative	0.00968
	DMFS mixed	ER+ LN negative tumors	0.01692
	DMFS mixed	Luminal B	0.02931
	RFS	Grade 3	0.04818
Snai1, Gli1	DMFS mixed	Grade 1	0.01909
	DMFS mixed	Luminal A	0.02437
	DMFS mixed	PAM50-Luminal A	0.0384
	RFS	Grade 1	0.01812
	RFS	-	0.041
Twist1, Gli1	DMFS	Basal	0.01376
	DMFS mixed	Basal	0.0268
	RFS	LN negative	0.05203

High expression of only both EMT-TFs *and GLI1* correlate with worsened prognosis across multiple breast subtypes and grades, while high expression of either the EMT-TF or *GLI1* alone does not. DMFS – Distant metastasis-free survival, RFS – Relapse-free survival, DMFS mixed – DMFS mixed with RFS data. Data obtained from GOBO or KM Plotter (in red).

Supplementary Table 2. Primer sequences for qRT-PCR analyses

Primer	Forward Primer (5'-3')	Reverse Primer (5'-3')
Six1	TGCGCCGAAAATTTCCA	TTGAAGCAGTAGCTGGTCTCC
Twist1	GGAGTCCGCAGTCTTACGAG	TCTGGAGGACCTGGTAGAGG
Snai1	GAAAGGCCTTCAACTGCAAA	TGACATCTGAGTGGGTCTGG
Gli1	GATGACCCCACCACCAATCA	AGAAAAGAGTGGGCCCTCGG
Ptch1	CTGGCTTCAGGGACTTCAGG	CGGTTTGCACCAGGAGTTTG
Ptch2	CCAGAGATCCTGAGTCCACC	GGCTGGATGGATGTAGGCAC
VEGF-A	AAGAAAATCCCTGTGGGCCTT	TTTCCTGGTGAGAGATCTGCA
Bcl-2	GGAGGCTGGGATGCCTTTGT	TTCACTTGTGGCCCAGATAGG
BMP-4	ACCGAATGCTGATGGTCGTT	CAGAAGTGTCGCCTCGAAGT
FoxF1	TCTCGCTCAACGAGTGCTTC	GTTCATCATGCTGTACATGGGC
CyclinD1	CTGCTCCTGGTGAACAAGC	TGTGGCACAGAGGGCAAC
MTSS1	AAGAACGTGGCCGATTCTGT	TGGACTTTCTGGACATGGTGG
SHH	GAAGAGGAGGCACCCCAAAA	CCCTTCATACCTTCCGCTGG
IHH	CCGCGACCGCAATAAGTATG	CGAGTGCTCGGACTTGACG
DHH	ACATCACTACGTCTGACCGC	ACCGCCAGTGAGTTATCAGC
GAPDH	CATCACCATCTTCCAGGAGC	ATGCCAGTGAGCTTCCCGTC
PP1B	GGAGATGGCACAGGAGGAAA	CGTAGTGCTTCAGTTTGAAGT
18S	GTAACCCGTTGAACCCCATT	CCATCCAATCGGTAGTAGCG

Supplementary Table 3. Antibodies used for Western blot analyses and ICC

Antibody	Company	Catalog Number	Dilution Used
SHH	Cell signaling	2207	1:1000 – WB
E-Cadherin	BD Biosciences	610181	1:2500 – WB<br>1:300 – ICC
E-Cadherin	Cell signaling	3195S	1:300 – ICC
Vimentin	BD Biosciences	550513	1:2500 – WB
Cytokeratin-18	Abcam	ab82254	1:10,000 – WB
Fibronectin	BD Biosciences	610077	1:500 – WB
Eya2	Sigma-Aldrich	HPA027024	1:1000
HDAC1	Santa Cruz	sc-7872	1:1000 – WB
β-tubulin	Sigma-Aldrich	T8328	1:5000 – WB
GAPDH	Cell signaling	2118S	1:1000 – WB
AlexaFluor 488 α-rabbit IgG	Invitrogen	A11008	1:300 – ICC
AlexaFluor 594 α-mouse IgG	Invitrogen	A11005	1:300 – ICC
HRP-anti-mouse IgG	Sigma-Aldrich	A9044	1:10,000 – WB
HRP-anti-rabbit IgG	Sigma-Aldrich	A9169	1:10,000 – WB

WB – Western blot analysis, ICC - Immunocytochemistry
